# Arctigenin from *Saussurea medusa* Maxim. Targets the PI3K/AKT Pathway to Inhibit Hepatocellular Carcinoma Proliferation and Induces Apoptosis

**DOI:** 10.3390/nu17193151

**Published:** 2025-10-02

**Authors:** Ruitao Yu, Jinghua Chen, Ruixue Yu

**Affiliations:** 1 Qinghai Provincial Key Laboratory of Tibetan Medicine Research, Northwest Institute of Plateau Biology, Chinese Academy of Sciences, Xining 810008, China; 2 University of Chinese Academy of Sciences, Beijing 101408, China; 3 School of Medicine, College of Pingdingshan University, Pingdingshan 467000, China

**Keywords:** arctigenin, hepatocellular carcinoma, PI3K/AKT pathway, network pharmacology, Tibetan medicine, apoptosis

## Abstract

Background: Hepatocellular carcinoma (HCC) is a highly lethal malignancy with limited therapeutic options. Arctigenin (ARC), a natural lignan derived from *Saussurea medusa*, exhibits anti-cancer activity, but its mechanism against HCC remain incompletely elucidated. Methods: This study integrated network pharmacology, molecular docking, molecular dynamics, in vitro, and in vivo experiments to investigate ARC’s anti-HCC effects. Results: Seventy-five potential targets shared between ARC and HCC were identified, with KEGG analysis highlighting the PI3K/AKT pathway as central. ARC showed strong binding to key proteins, and molecular dynamics indicated stable interactions with PIK3CA and GSK3B. In HepG2 cells, ARC inhibited proliferation in a dose- and time-dependent manner (IC50: 11.17 μM at 24 h, 4.888 μM at 48 h), induced apoptosis at high concentrations, suppressed PIK3CA phosphorylation, and increased GSK3B (Ser9) phosphorylation. In H22 tumor-bearing mice, ARC dose-dependently inhibited tumor growth (high dose: 50.6% vs. 63.0% for CTX) with minimal weight loss. Conclusions: These findings suggest ARC suppresses HCC by modulating the PI3K/AKT pathway, providing evidence for its development as a plant-derived therapeutic agent.

## 1. Introduction

Hepatocellular carcinoma (HCC) is a prevalent and lethal malignancy worldwide [1], ranking fourth in incidence and second in mortality among males in China, as per the 2022 IARC Global Cancer Burden data. Early diagnosis is challenging due to subtle symptoms, leading to rapid progression and poor prognosis in advanced stages, with a five-year survival rate remaining low [2]. Current treatments include surgery, chemotherapy, radiotherapy, immunotherapy, and targeted drugs such as angiogenesis inhibitors and TACE [3]. Identifying key therapeutic targets and developing low-toxicity drugs hold promise for improved treatment options.

Natural compounds from herbs are valuable therapeutic sources. *Saussurea medusa* Maxim. (SMM) is a plant distributed in regions like Qinghai and Tibet [4]. Traditional Tibetan medicine uses SMM for conditions such as head trauma, pain, and stroke. Modern studies reveal its anti-cancer, anti-inflammatory, and antioxidant properties, among others [5]. Our team has identified over 160 compounds from SMM, including arctigenin (ARC), a lignan with anti-inflammatory, antioxidant, and anti-cancer properties. ARC demonstrates significant potential for anti-tumor drug development.

Pharmacokinetic studies have further revealed that ARC is rapidly absorbed and widely distributed in organs such as the liver, intestine, kidney, and pancreas, with relatively high oral bioavailability in animal models [6]. However, it undergoes extensive metabolic transformations including glucuronidation and hydrolysis in the liver, intestine, and plasma, which may hinder its in vivo efficacy after oral administration [7]. Notably, a Phase I clinical trial of an ARC-enriched extract in pancreatic cancer patients still reported good oral availability, favorable safety, and preliminary efficacy [8]. Collectively, these findings suggest that although ARC is subject to metabolic modification, it retains pharmacokinetic and safety characteristics compatible with therapeutic application.

Anti-cancer effects of ARC are documented in various cancers, including HCC [9], prostate cancer, breast cancer, and melanoma. Its mechanisms involve pathways like MAPK/AP-1, and TGF-β/Smad, leading to apoptosis, autophagy inhibition, and metastasis suppression [10,11,12]. While ARC shows selective toxicity against HCC cells and minimal impact on normal liver cells [11], its specific targets remain unclear. Studies suggest ARC acts via pathways such as PI3K/AKT inhibition, P53 protein accumulation, and ROS production [13,14]. It also inhibits EMT and metastasis via GSK3B-dependent β-catenin signaling, and regulates fibrosis through the TGF-β/Smad pathway [10].

This study explores anti-HCC mechanisms of ARC through network pharmacology, molecular docking, and dynamics simulations, validated by in vitro and in vivo experiments to provide deeper insights into its therapeutic potential.

## 2. Materials and Methods

### 2.1. Materials

#### 2.1.1. Cell

Human HCC cells (HepG2) and Murine Hepatocellular Carcinoma Cells (H22) were purchased from the Cell Resource Center, Shanghai Institutes for Biological Sciences, Chinese Academy of Sciences.

#### 2.1.2. Reagents and Instruments

**Figure A1:**
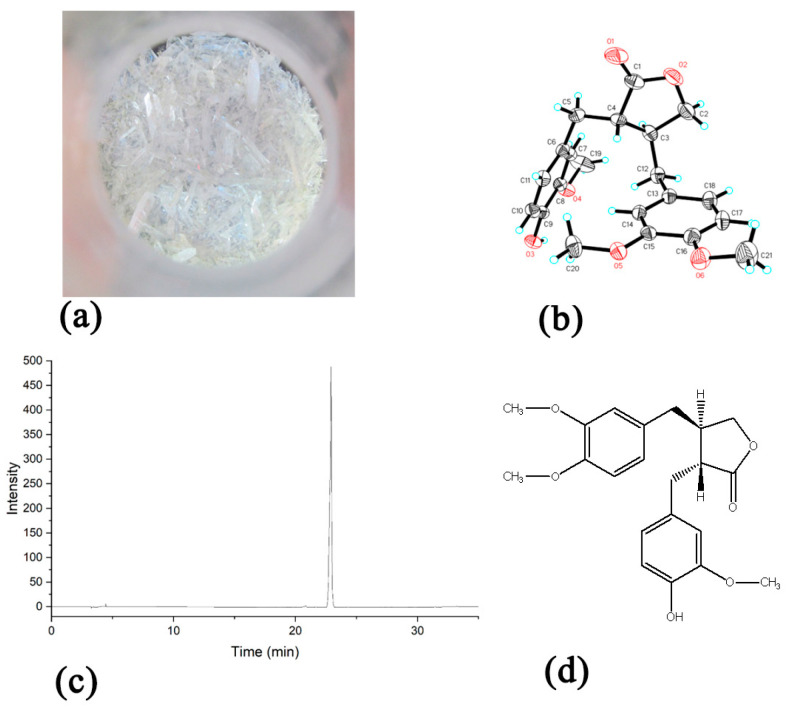
Structural Characterization and Purity Analysis of ARC. (**a**) Single Crystal Diagram. (**b**) Single Crystal X-ray Diffraction. (**c**) HPLC purity spectrum of ARC. (**d**) Structure of ARC.

The reference compound ARC was obtained from the previous isolation results of our research group, with a purity of >98% as confirmed by High Performance Liquid Chromatography (HPLC, see Figure A1, acetonitrile gradient elution, aqueous phase containing 0.2‰ formic acid, runtime of 35 min, Shimadzu Essentia LC-16 system, Megres column). The plant material was collected in August 2018 from Yeniu Valley, Qilian County, Qinghai Province, China, at an altitude of 4100 m. The specimen is preserved in the Herbarium of the Northwest Institute of Plateau Biology, Chinese Academy of Sciences (No. 0341202).

Simvastatin reference compound (batch number 24240515002, purity >99%) were purchased from Beijing Solarbio Science & Technology Co., Ltd. (Beijing, China); Glyceraldehyde-3-phosphate dehydrogenase (GAPDH) antibody (batch number BST17383876), GSK3B antibody (batch number 24F107311F17), mechanistic target of rapamycin (mTOR) antibody (batch number 24F114106F24), Phosphatidylinositol-4,5-bisphosphate 3-kinase catalytic subunit alpha (PIK3CA) antibody (batch number BOS6238BP2883), pGSK3B (Ser9) antibody (batch number 24F087204A08) were all purchased from Wuhan Boster Biological Technology Co., Ltd. (Wuhan, China); metformin reference compound (batch number L396024, purity >97%) was purchased from Shanghai Haohong Biomedical Technology Co., Ltd. (Shanghai, China); LY294002 reference compound (batch number A825051337769) was purchased from APExBIO Technology LLC. (Houston, TX, USA); pPIK3CA (Tyr317) antibody (batch number 2i44771) and pmTOR (Ser248) antibody (batch number 8g6448) were purchased from Affinity Biosciences; Cyclophosphamide reference compound (batch number H32020857) was purchased from Jiangsu Hengrui Medicine Co., Ltd. (Lianyungang, China) Model 680 microplate reader, PowerPac Basic electrophoresis unit, and electrophoresis/transfer system were obtained from Bio-Rad (Hercules, CA, USA); The FlowSight flow cytometer was purchased from Cytek^®^ Biosciences Inc. (Fremont, CA, USA).

#### 2.1.3. Experimental Animals

This study was performed on female ICR mice aged 4–5 weeks. All mice were housed in a controlled environment free from specific pathogens. They were subjected to a standard light–dark cycle, and ad libitum access to food and water. All animal experimental procedures were approved by the Institutional Animal Care and Use Committee (IACUC) of Nanjing Rameda Pharmaceutical Co., Ltd., (Nanjing, China) (Permit No. IACUC-2021020107, approved on 2 February 2021). All procedures adhered to the ARRIVE guidelines and were conducted in accordance with the UK Animals (Scientific Procedures) Act 1986 and relevant guidelines, the EU Directive 2010/63/EU on the protection of animals used for scientific purposes, or the US National Research Council’s Guide for the Care and Use of Laboratory Animals. To ensure animal welfare, efforts were made to minimize both the number of animals used and their discomfort. Humane endpoints were strictly observed, including tumor volume not exceeding 10% of body weight (based on equivalent volume conversion), body weight loss reaching 20–25%, and other clinical signs such as loss of appetite. Euthanasia was performed by CO_2_ inhalation in accordance with AVMA guidelines.

### 2.2. Network Pharmacology

The predicted targets of ARC were identified using the SEA, Super-PRED, and SwissTargetPrediction databases [15]. HCC-related targets were obtained from DisGeNet, GeneCards, OMIM, and TTD databases. After consolidating and deduplicating results, overlapping targets of ARC and HCC were determined using a Venn diagram tool, representing potential anti-HCC targets [16].

Subsequently, the potential targets were analyzed in the STRING database to construct a protein interaction network [17]. The data were imported into Cytoscape 3.9.1, and key targets were identified using CytoNCA plugin [18].

Following this, the key targets were analyzed on the Bioinformatics platform for GO and KEGG enrichment. KEGG pathways were classified using the “Pathway Secondary Classification Summary” tool [19], and key classifications were visualized. GO analysis focused on the CC, MF, and BP categories and was visualized accordingly.

Finally, the potential targets, GO, and KEGG results were organized to establish drug–target–pathway relationships. A regulatory network for ARC against HCC was constructed in Cytoscape, and core targets were identified by ranking nodes based on Degree values from Network Analysis [20].

### 2.3. Molecular Docking

To investigate the molecular interactions between ARC and core targets, the ARC structure was downloaded from the PubChem database and energy-minimized using Chem3D. The top 12 targets ranked by Degree were selected, and their high-resolution structures were retrieved from the PDB database. Protein and ligand preparations were performed using Swiss-PdbViewer 4.1.0, PyMOL 2.5.0, and AutoDockTools 1.1.2 [21]. Binding sites were predicted with DeepSite tool on the PlayMolecule platform (www.playmolecule.com, accessed on 28 June 2024) [22], and molecular docking was conducted using AutoDock Vina, with visualizations generated in Discovery Studio 2021.

### 2.4. Molecular Dynamics (MD) Simulation

Receptor-ligand complexes with lower binding energies were further analyzed via molecular dynamics (MD) simulation using Gromacs with the CHARMM36 force field, while ligand parameters were generated using Sobtop tool (Tian Lu, Sobtop, version 1.0 (dev3.1), http://sobereva.com/soft/Sobtop, accessed on 15 August 2024) [23,24]. Simulations ran for 50 ns, and RMSD curves were analyzed to assess binding stability. Stable MD segments were used for energy calculations with the gmx_mmpbsa script (DOI 10.5281/zenodo.6408973), and binding effects were evaluated accordingly.

### 2.5. In Vitro Studies

In the in vitro experiments, HepG2 cells were cultured under standard conditions [25,26]. HepG2 cells were divided into the control group, positive controls (Metformin, Simvastatin, and LY294002), and ARC treatment groups at low, medium, and high concentrations. A wide concentration range (0.39–400 μM) was initially used to generate a dose–response curve and determine the IC50 values. Subsequently, representative doses, corresponding to below, around, and above the IC50, were selected for further mechanistic studies.

#### 2.5.1. MTT Assay for Cell Inhibition

Log-phase HepG2 cells were seeded in 96-well plates with five replicates per group. After adherence, cells were treated with medium containing varying concentrations of ARC (0.39–400 μmol/L) for 24 or 48 h. Control wells received drug-free medium, and blank wells (no cells) served as baselines. After treatment, cell viability and IC50 values were determined using the MTT assay according to the manufacturer’s protocol.

#### 2.5.2. Flow Cytometry for Apoptosis Detection

Metformin (Met, 10 mM) [27] and simvastatin (Sim, 19 μM) [28] served as positive controls. ARC was tested at low (LA, 3 μM), medium (MA,12 μM), and high (HA, 48 μM) concentrations. An additional group included 10 μM LY294002 combined with medium ARC concentration (MA + LY) [29].

Apoptosis was assessed using Annexin V-FITC/PI staining following the manufacturer’s protocol and analyzed by flow cytometry.

#### 2.5.3. Western Blotting Analysis of Protein Expression

Cells were treated as described in Section 2.5.1. After 24 h, cells were collected, and total protein was extracted. Protein concentrations were determined using the BCA method. Western blotting analysis was carried out as reported in the literature [30], with protein loading, transfer, antibody incubation, and imaging steps.

### 2.6. In Vivo Studies

In the in vivo experiments, H22 tumor-bearing mice were randomly divided into the control group (NS), positive control group (CTX), and ARC treatment groups at low, medium, and high doses. The main observation indicators included body weight, tumor volume, tumor weight, and tumor inhibition rate.

#### 2.6.1. Preparation of the Model

The cultured H22 ascites cells were harvested and diluted with physiological saline at a 1:8 ratio. A 0.1 mL aliquot of the suspension was injected subcutaneously into the right axilla of each mouse.

#### 2.6.2. Grouping and Administration

Tumor diameters were measured with a vernier caliper, and when the volume reached approximately 50–100 mm^3^, thirty mice were randomly allocated into five groups (*n* = 6 per group). The groups included the model control (NS), positive control (Cyclophosphamide, CTX), and three ARC treatment groups at different dose levels. Each treatment was given once daily for 14 consecutive days (Table 1). ARC solutions were prepared in CMC-Na and administered by gavage, while CTX was dissolved in normal saline and given intraperitoneally.

#### 2.6.3. Observation Indicators

The antitumor effects of the test samples were evaluated dynamically by measuring tumor diameters. At the end of the administration period, mice were euthanized, and tumors were surgically excised and weighed. Tumor volume (TV), relative tumor volume (RTV), relative tumor proliferation rate (T/C), and tumor growth inhibition rate were then calculated.

### 2.7. Statistical Analysis

Statistical analyses were performed using GraphPad Prism 9. One-way and two-way ANOVA were applied for comparisons among multiple groups [31]. *p* < 0.05 was considered statistically significant.

## 3. Results

### 3.1. Network Pharmacology Results

**Figure 1:**
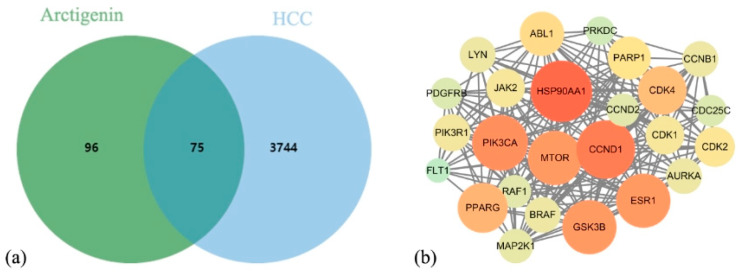
Intersection of ARC Targets with HCC Targets and Identification of Key Targets. (**a**) ARC targets against HCC. (**b**) PPI network of core targets.

After merging and deduplicating results from databases, 171 ARC targets and 3819 HCC targets were identified. The intersection of these datasets revealed 75 potential ARC targets against HCC (Figure 1a). A PPI network analysis of these potential targets identified 25 key targets based on centrality indicators above the median values (Figure 1b). In the network visualization, node size corresponds to degree values, and nodes are color-coded from green to red, highlighting the critical roles of these highly connected targets.

**Figure 2:**
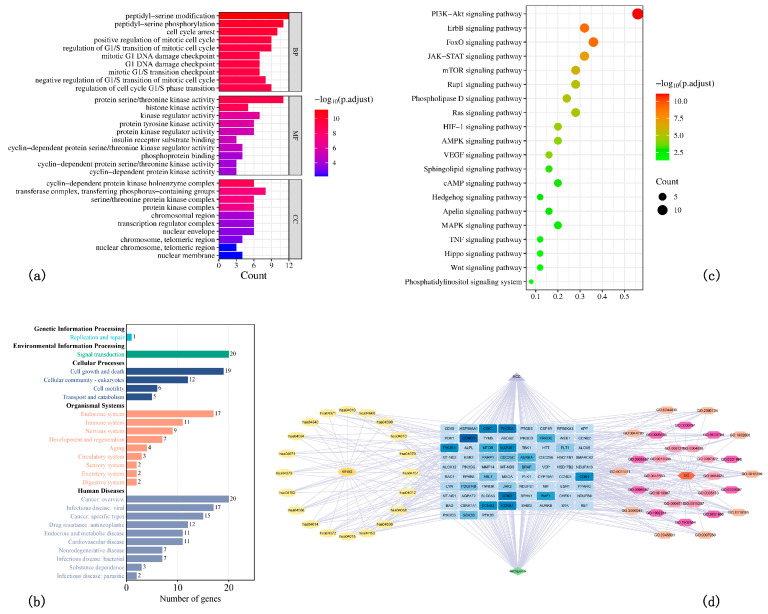
GO and Pathway Enrichment Analysis Results and Overall Network Diagram. (**a**) Top enriched GO terms for biological processes, cellular components, and molecular functions. (**b**) KEGG pathways grouped by secondary categories. (**c**) Bubble chart of enriched pathways, highlighting PI3K/AKT and related signaling pathways. (**d**) Drug–target–pathway network integrating GO and KEGG results.

GO enrichment analysis identified 1387 biological processes (BPs), 65 cellular components (CCs), and 108 molecular functions (MFs). The primary BPs included modifications and phosphorylation of peptidyl-serine, cell cycle arrest, and regulation of the mitotic cell cycle. Important MFs involved protein serine/threonine kinase activity and histone kinase activity (Figure 2a). These findings suggest that ARC primarily exerts its anti-HCC effects through the regulation of kinase activity and cell cycle processes.

KEGG enrichment analysis revealed ARC targets were enriched in 98 signaling pathways across 25 secondary classifications, with key pathways like PI3K/AKT, FoxO, ErbB, and JAK-STAT signaling identified within the “Signal transduction” category (Figure 2b). A bubble chart highlights these pathways’ significant roles in anti-HCC effects of ARC (Figure 2c).

Figure 2d illustrates the drug–target–pathway relationships. Yellow nodes on the left represent key pathways, three rings on the right show GO categories, and the central rectangles’ color intensity indicates target significance.

### 3.2. Molecular Docking Results

**Figure 3:**
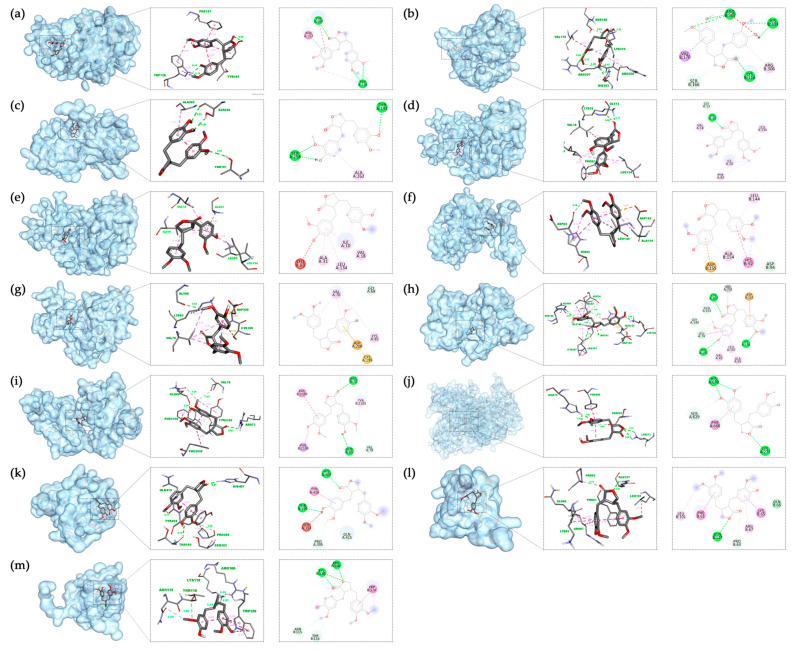
Molecular Docking Results of ARC and core target proteins: (**a**) AURKA, (**b**) CCNB1, (**c**) CCND1, (**d**) CDK1, (**e**) CDK2, (**f**) CDK4, (**g**) GSK3B, (**h**) MAP2K1, (**i**) MTOR, (**j**) PIK3CA, (**k**) PIK3R1, (**l**) RAF1, (**m**) BRAF.

From the network analysis, the top 13 targets with the highest degree values were selected for molecular docking. Binding affinities were categorized as follows: values below −4.25 kcal/mol indicate binding activity, and below −7 kcal/mol reflect strong interactions [32]. Docking results showed that all 13 core targets had binding energies below −4.25 kcal/mol with ARC, with mTOR and CDK1 exhibiting the lowest affinities, both below −8 kcal/mol, indicating exceptionally strong interactions (Table 2). These findings suggest favorable interactions between ARC and the core targets (Figure 3). Binding energies of the thirteen targets and ARC were visualized (Figure 3a–m), showing hydrogen bonds and other key interactions between ARC and the targets.

### 3.3. Molecular Dynamics Simulation Results

**Figure 4:**
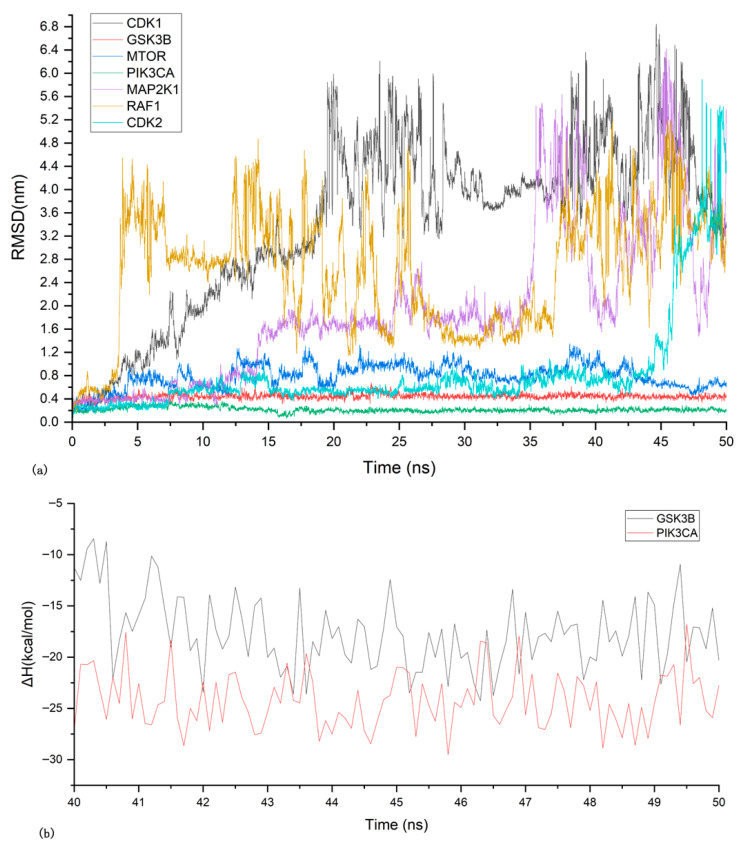
Molecular Dynamics Simulation Results. (**a**) RMSD profiles of protein–ligand complexes over 50 ns simulations. (**b**) Binding energy profiles (enthalpy changes) of GSK3B and PIK3CA complexes.

Based on molecular docking results, the ARC complexes with CDK1, mTOR, GSK3B, PIK3CA, MAP2K1, CDK2, and RAF1 were subjected to 50 ns molecular dynamics simulations to evaluate structural stability. RMSD curves showed that only GSK3B and PIK3CA remained relatively stable (Figure 4a) with smaller RMSF values, meeting the stability criterion of RMSD fluctuations below 0.2 nm [33].

Binding energy calculations for GSK3B and PIK3CA demonstrated stable binding energies (enthalpy changes) (Figure 4b), suggesting reliable interactions. Energy decomposition analysis (Table 3) revealed that van der Waals forces contributed significantly to binding, with PIK3CA showing a lower total binding energy than GSK3B.

### 3.4. In Vitro Results

#### 3.4.1. ARC Inhibits HepG2 Proliferation

**Figure 5:**
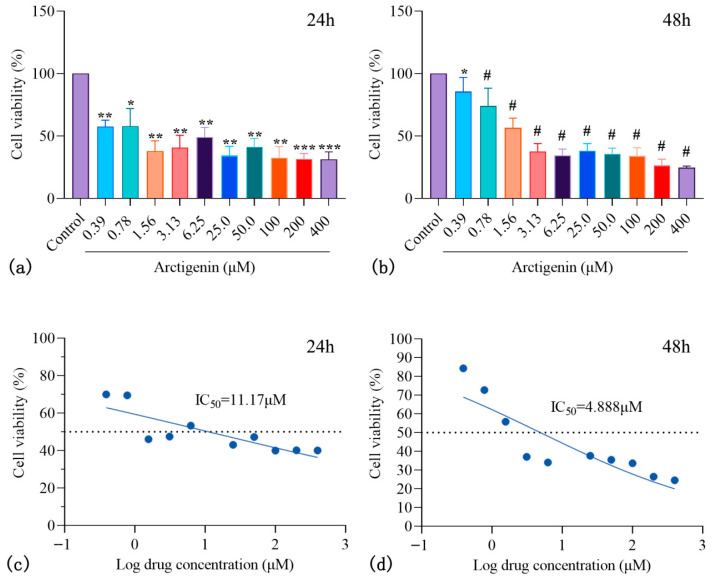
The effect of ARC on HepG2 cell proliferation. (**a**,**b**) Cell viability of HepG2 cells treated with different concentrations of ARC for 24 h (**a**) and 48 h (**b**). (**c**,**d**) Dose–response curves and calculated IC50 values at 24 h (**c**) and 48 h (**d**). ARC significantly inhibited HepG2 proliferation in a dose-dependent manner compared to the control group. Note: Compared to the control group: * *p* < 0.05, ** *p* < 0.01, *** *p* < 0.001, # *p* < 0.0001.

The results show that, compared to the control group, ARC at various concentrations significantly inhibited HepG2 cell viability after both 24 h (Figure 5a) and 48 h (Figure 5b). The IC50 values were 11.17 μM (Figure 5c) and 4.888 μM (Figure 5d), respectively. This suggests that ARC may exert its anti-HCC effects by inhibiting cell proliferation.

#### 3.4.2. The Effect of ARC on HepG2 Cell Apoptosis

**Figure 6:**
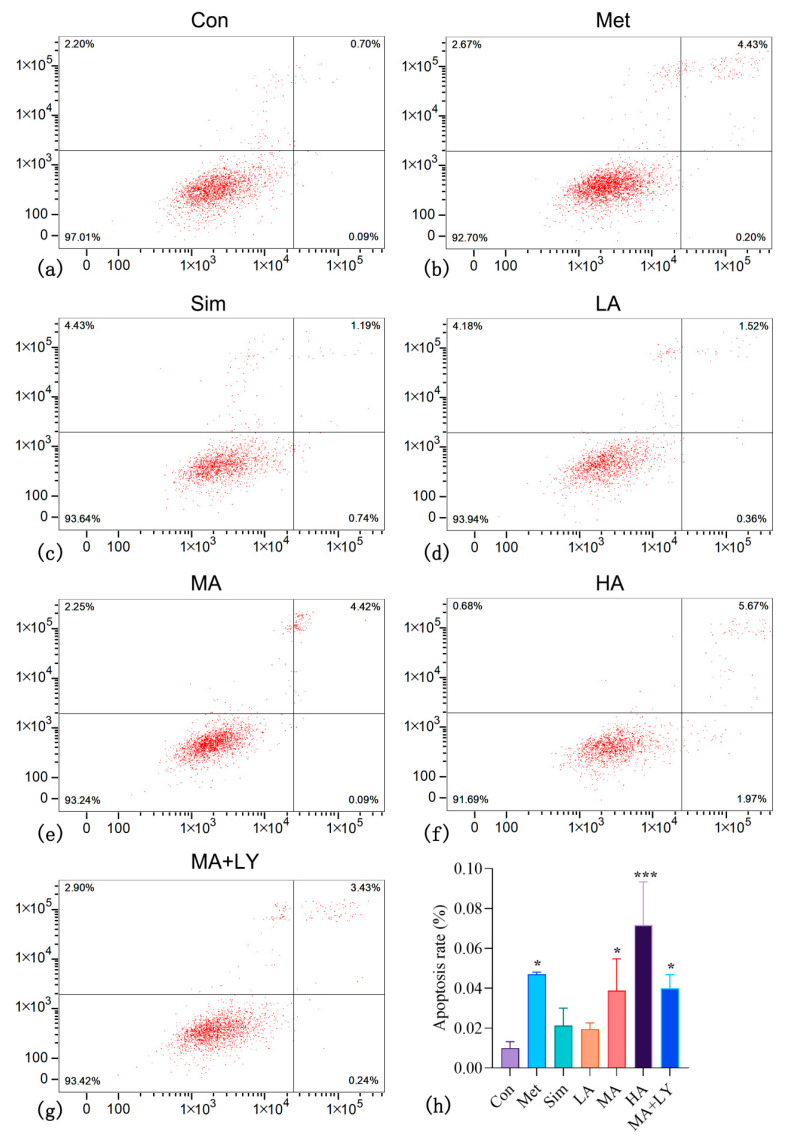
The Effect of ARC on HepG2 Cell Apoptosis. (**a**–**g**) Flow cytometry analysis showing apoptosis rates in HepG2 cells treated with low (LA), medium (MA), and high (HA) concentrations of ARC, as well as Met, Sim and MA + LY groups. (**h**) Comparison of apoptosis rates. Note: Compared to the control group: * *p* < 0.05, *** *p* < 0.001.

Flow cytometry results (Figure 6a–g) showed a significant increase in HepG2 apoptosis rates in the Met, MA, and HA groups compared to the control (*p* < 0.05 and *p* < 0.001), with higher ARC concentrations further enhancing apoptosis. No significant difference was observed between the MA and MA + LY groups (*p* > 0.05) (Figure 6h). Apoptosis in the Met group was between the MA and HA groups, while the Sim group fell between LA and MA, closer to LA.

#### 3.4.3. The Effect of ARC on the Expression of PI3K/AKT Pathway-Related Proteins in HepG2

**Figure 7:**
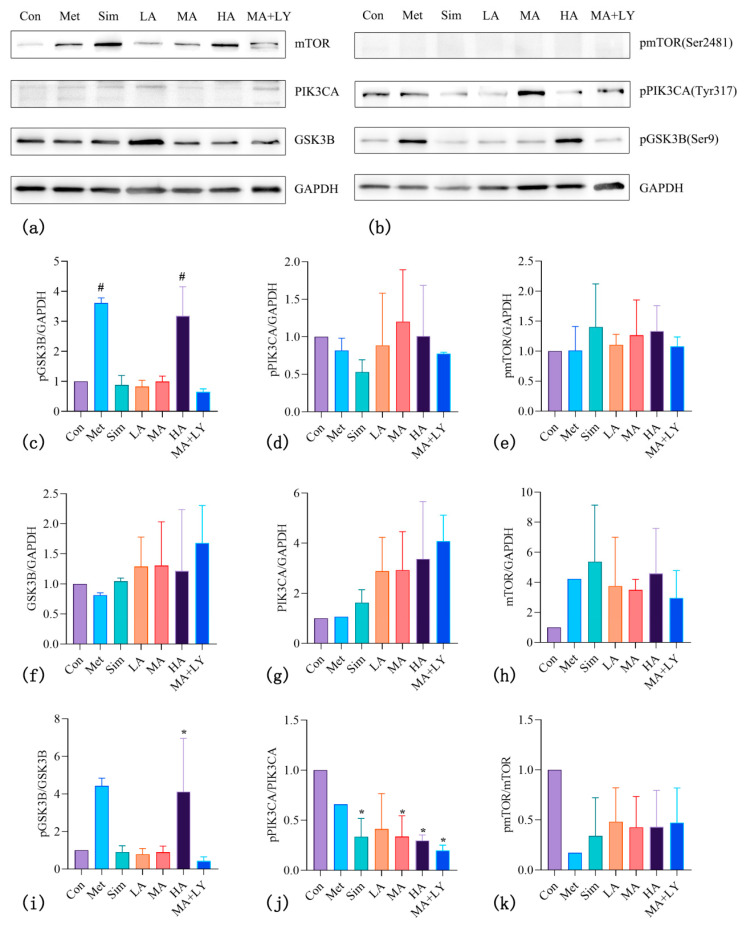
The Effect of ARC on GSK3B/PIK3CA/mTOR and their Phosphorylated Protein Expression. (**a**,**b**) Representative Western blotting bands of mTOR, pmTOR (Ser2481), PIK3CA, pPIK3CA (Tyr317), GSK3B, pGSK3B (Ser9), and GAPDH (loading control). (**c**–**k**) Quantitative analysis of the relative expression or phosphorylation ratio (phosphorylated/total protein) of these proteins in different treatment groups (Con, Met, Sim, LA, MA, HA, MA + LY). Note: Compared to the control group: * *p* < 0.05, # *p* < 0.0001.

Network pharmacology results indicate that the PI3K/AKT pathway may be a key mechanism underlying anti-HCC effects of ARC. Western blotting analysis was conducted to evaluate the expression of PI3K/AKT-related and downstream pathway proteins (Figure 7).

The phosphorylation levels (pGSK3B/total GSK3B) were significantly elevated in the Met and HA groups, indicating that both metformin (a positive control) and high ARC concentrations exert a similar regulatory trend in increasing GSK3B phosphorylation (*p* < 0.0001)—this consistency suggests that ARC, at high doses, can achieve a comparable effect to metformin in modulating this downstream protein of the PI3K/AKT pathway. No significant changes were observed in other groups (including LA, MA, Sim, and MA + LY groups).

The most prominent bands for pPIK3CA and PIK3CA appeared near the 180 kDa marker, which was higher than the molecular weight of PIK3CA (110 kDa). The mean PIK3CA expression in all ARC-treated groups exceeded that of the control, whereas the pPIK3CA/PIK3CA ratio exhibited a continuous dose-dependent decline from LA to MA to HA, indicating significant inhibition of PIK3CA phosphorylation (*p* < 0.05). The elevated PIK3CA expression may reflect an accumulation due to suppressed phosphorylation. From the perspective of positive controls, the Met group did not exhibit a significant inhibitory effect on PIK3CA phosphorylation, whereas the Sim group showed an inhibitory effect on PIK3CA phosphorylation that was comparable to that of the MA group. Compared with MA, the MA + LY group showed a further but non-significant decrease in the pPIK3CA/PIK3CA ratio (*p* > 0.05), indicating that LY294002 did not further enhance inhibition of PIK3CA phosphorylation.

The pmTOR bands were not clearly visible, while the total mTOR expression was higher in positive controls and ARC-treated groups than in the control group. The phosphorylation/total protein ratio indicated a reduction in mTOR phosphorylation in the treated groups, although the change was not statistically significant.

These findings suggest that ARC suppresses HCC proliferation via the PI3K/AKT signaling pathway, particularly by inhibiting the phosphorylation of PIK3CA, thereby preventing activation of these proteins and inducing apoptosis in HepG2 cells.

### 3.5. In Vivo Results

#### 3.5.1. Effect on the Body Weight of Test Animals

**Figure 8:**
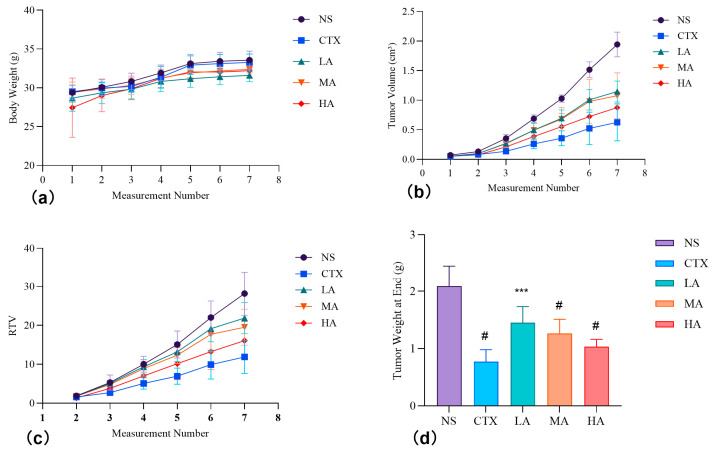
Effects of the Tested Samples on Body Weight and Tumor Volume in Mice with H22 Cells. (**a**) Dynamic alterations in body weight across measurement intervals. (**b**) Temporal changes in tumor volume during the experiment. (**c**) Variation in relative tumor volume (RTV). (**d**) Tumor weight at the experiment’s conclusion. Note: Compared to the control group: *** *p* < 0.001, # *p* < 0.0001.

**Figure A3:**
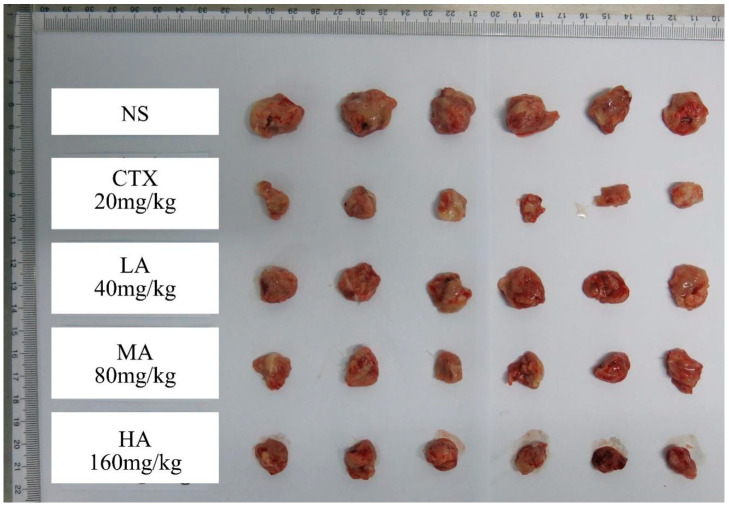
Tumor Samples Extracted from Mice.

Body weight data (Figure 8a, Figure A3, Table A1) showed that the CTX and ARC groups had lower mean body weights than the NS group, except at the first measurement, but most differences were not statistically significant (*p* > 0.05). Notably, the LA group exhibited significantly lower body weight than the NS group at the fifth to seventh measurements (*p* < 0.05). The HA group also showed significantly lower body weight than the NS group at the first measurement (*p* < 0.05), but this difference was not observed in subsequent measurements, indicating a normal growth trend. These results suggest that ARC had no obvious effect on body weight gain in mice.

#### 3.5.2. Changes in Tumor Volume and Weight, and Tumor Inhibition Effects

Tumor volume data (Figure 8b, Figure A3, Table A2) showed significant slowing of tumor growth in the CTX and HA groups starting from the fourth measurement (*p* < 0.0001 and *p* < 0.01). Significant differences between all treatment groups and the NS group appeared from the fifth measurement onward (*p* < 0.0001 or *p* < 0.01). Relative tumor volume analysis (Figure 8c, Table A3) revealed significant differences between the CTX and NS groups as early as the fourth measurement (*p* < 0.05). Differences in the HA, MA, and LA groups were observed at the fifth, sixth, and seventh measurements, respectively. The CTX group showed the lowest tumor proliferation rate (42.2%), while the LA group had the highest (77.6%) (Table 4).

At the experiment’s end, tumor weights were significantly lower in all treatment groups compared to the NS group (*p* < 0.0001 or *p* < 0.001), with the CTX group having the lowest tumor weight (Figure 8d). The CTX group also exhibited the highest tumor inhibition rate (63.0%), followed by HA (50.6%), MA (39.6%), and LA (30.56%) (Table 4).

Overall, these findings indicate that ARC inhibited tumor growth in a dose-dependent manner, with the strongest effect observed in the HA group, though still less pronounced than CTX.

## 4. Discussion

HCC is a highly aggressive and chemoresistant cancer with a significant global burden. Chinese medicine, including natural compounds like arctigenin (ARC), is explored for its potential in treating HCC. This study investigated the molecular mechanisms of ARC in HCC through network pharmacology, molecular docking, molecular dynamics simulations, and in vitro and in vivo experiments.

Network pharmacology identified 75 common targets between ARC and HCC, with 25 key targets showing high centrality in the protein–protein interaction (PPI) network. GO and KEGG analyses highlighted the PI3K/AKT, FoxO, ErbB, and JAK-STAT pathways. Molecular docking and dynamics simulations indicated strong binding between ARC and key targets, with stable interactions in the ARC-PIK3CA and ARC-GSK3B complexes.

In this study, ARC inhibited HepG2 cell viability in a dose- and time-dependent manner. The IC50 values at 24 h and 48 h indicated that cellular sensitivity to ARC increased with prolonged exposure. Notably, at higher concentrations, the decline in cell viability reached a plateau. This phenomenon is consistent with the pharmacodynamic characteristics of targeted inhibitors [34], where inhibition of relevant targets reaches a maximum near the IC50 level, and further dose escalation does not enhance efficacy [35]. Under high-dose conditions, a proportion of cells remained viable, which may be attributable to dormant populations or cells with active drug efflux capacity, as documented in cancer stem cell studies [36,37]. Conversely, at lower doses, cell viability decreased sharply with increasing concentrations, reflecting the dose-dependent inhibitory effect. Remarkably, within this range, cell viability at 48 h was slightly higher than at 24 h under the same concentrations, which may be attributed to drug metabolism or degradation, cellular adaptive responses, or minor experimental variability.

Consistent with these observations, ARC induced apoptosis in HepG2 cells in a concentration-dependent manner. Western blotting analysis subsequently revealed that ARC increased GSK3B phosphorylation while inhibiting PIK3CA phosphorylation, supporting its anticancer effects through modulation of the PI3K/AKT pathway. However, co-treatment with the PI3K inhibitor LY294002 did not further enhance apoptosis, possibly due to competition between ARC and LY294002 for binding PIK3CA.

Anticancer mechanisms of ARC vary across tumor types. It affects the MAPK/AP-1, AKT/NF-κB [12], and GM-CSF/TSLP/STAT3/β-catenin pathways [38] in breast cancer, and induces apoptosis in ovarian and prostate cancer cells through ROS/p38 MAPK [13] and PI3K/AKT/mTOR [14] pathways. In HepG2 cells, ARC inhibits the PI3K/AKT pathway by reducing PIK3CA phosphorylation.

Molecular dynamics simulations revealed strong binding interactions between GSK3B and ARC. GSK3B is a serine/threonine kinase involved in key cellular processes such as proliferation, survival, gene expression, and development. Its activity is controlled by phosphorylation at Ser9 (inactive) and Tyr216 (active). Inactivation of GSK3B through phosphorylation at Ser9 leads to decreased degradation of β-catenin [39], promoting tumor cell proliferation.

Interestingly, our data showed that high doses of ARC increased GSK3B phosphorylation at Ser9, whereas medium and low doses did not. This apparent paradox—enhanced Ser9 phosphorylation concurrent with PI3K/AKT inhibition—suggests that ARC may induce Ser9 phosphorylation through PI3K/AKT-independent mechanisms. Potential alternative regulators include SIRT2-mediated deacetylation or Wnt-related signaling, which can modulate GSK3B activity without relying on classical AKT activation. Furthermore, ARC treatment did not significantly affect the cell cycle of HCC cells [9], indicating that its pro-apoptotic effects may involve other GSK3B-associated pathways or additional mechanisms beyond the canonical PI3K/AKT/GSK3B axis.

Our data showed that high doses of ARC increased GSK3B phosphorylation at Ser9, while medium and low doses did not. However, ARC treatment did not significantly affect the cell cycle of HCC cells [9], indicating that ARC may impact other pathways associated with GSK3B, and apoptosis likely occurs through mechanisms beyond the PI3K/AKT/GSK3B pathway.

Molecular docking identified mTOR as a key target with strong binding to ARC. mTOR is a serine/threonine kinase regulating cell growth and metabolism [40]. In HCC, the mTOR pathway is upregulated [41], contributing to tumor growth and spread. Although our Western blotting results did not show a statistically significant reduction in mTOR phosphorylation upon ARC treatment, the strong predicted binding suggests that ARC may still interact with mTOR, and the lack of observable effect could be due to the limited sensitivity of our detection method for phosphorylated mTOR.

Moreover, the observed tumor inhibition in vivo, where ARC displayed a dose-dependent reduction in H22 tumor growth, provides further support for its therapeutic potential. These in vivo results align with the identified molecular mechanisms.

The results of this study demonstrate that the anticancer effects of ARC on HCC cells are associated with apoptosis. The pro-apoptotic activity of ARC is mediated through the inactivation of the PI3K/AKT signaling pathway. These findings suggest that ARC could be an effective treatment for HCC, particularly in cases with abnormally activated PI3K/AKT signaling. Due to its biosafety and efficiency, ARC holds significant research and application potential as an anti-HCC drug.

## 5. Conclusions

In this study, network pharmacology identified 75 potential anti-HCC targets of ARC, with enrichment pointing to the PI3K/AKT pathway. Molecular docking and dynamics simulations suggested stable binding of ARC to key targets, especially PIK3CA and GSK3B. In vitro, ARC significantly inhibited HepG2 cell proliferation and induced apoptosis, accompanied by suppressed phosphorylation of PIK3CA, and enhanced phosphorylation of GSK3B at Ser9. In vivo, ARC showed dose-dependent tumor growth inhibition in H22 tumor-bearing mice. These findings suggest that ARC exerts anti-HCC effects primarily through modulation of the PI3K/AKT pathway and may serve as a promising candidate for further development of plant-based therapeutics against HCC.

While this study focused on HepG2 cells and the H22 mouse model, and primarily on the PI3K/AKT pathway, further mechanistic exploration in animal models would complement the in vitro findings and provide a more comprehensive understanding of ARC’s anti-HCC effects. Moreover, pharmacokinetic profiles and comprehensive safety assessments of ARC remain to be further investigated. Additional experiments evaluating its toxicity in normal hepatocytes and in vivo models, particularly at concentrations comparable to or higher than those of CTX, would help to clarify its effective and safe dose range. Together, these directions would provide valuable evidence to support the future clinical translation of ARC as a plant-derived anti-HCC agent.

## Figures and Tables

**Table 1:** Grouping and Dosage of Administration.

Group	Administration Route	Dose (mg/kg)	Volume Per Day (ml)
NS	Gavage	-	0.1 mL/10 g
CTX	Intraperitoneal	20	0.1 mL/10 g
LA	Gavage	40	0.1 mL/10 g
MA	Gavage	80	0.1 mL/10 g
HA	Gavage	160	0.1 mL/10 g

**Table 2:** Selection of Receptor Structures and Docking Affinity Results.

No.	Receptor	Degree of Receptor	Structure	Affinity (kcal/mol)
1	CCND1	28	6P8E	−5.726
2	PIK3CA	24	4JPS	−7.55
3	CDK2	24	6Q4G	−6.569
4	CDK1	23	6GUK	−8.151
5	CCNB1	22	6GU2	−5.78
6	MAP2K1	21	3VVH	−6.683
7	CDK4	21	6P8E	−5.967
8	PIK3R1	21	5GJI	−5.96
9	RAF1	18	6VJJ	−6.269
10	mTOR	16	4DRI	−8.309
11	AURKA	15	5OS0	−6.143
12	GSK3B	11	1Q5K	−7.116
13	BRAF	11	8JNA	−5.562

**Table 3:** Binding Energy Contribution Decomposition (kcal/mol).

Complex	Contribution							
	ΔG	−TΔS	ΔH	MM	PB	SA	COU	VDW
GSK3B	−5.30	12.35	−17.66	−52.39	40.71	−5.98	−15.03	−37.35
PIK3CA	−19.53	4.68	−24.21	−50.55	32.18	−5.84	−8.65	−41.90

**Table 4:** Summary of In Vivo Experiment Results.

Group	Start of Experiment			End of Experiment			Relative Tumor Proliferation Rate (T/C)	Tumor Weight at End (g)	Tumor Inhibition Rate (%)
	Number of Animals	Body Weight (g)	Tumor Volume (cm^3^)	Number of Animals	Body Weight (g)	Tumor Volume (cm^3^)			
NS	6	29.4 ± 1.0	0.072 ± 0.021	6	33.6 ± 1.2	1.944 ± 0.210	-	2.09 ± 0.36	-
CTX	6	29.5 ± 0.8	0.052 ± 0.010	6	33.3 ± 1.1	0.626 ± 0.312 #	42.2%	0.77 ± 0.21 #	63.0%
LA	6	28.7 ± 1.7	0.053 ± 0.010	6	31.6 ± 0.8 *	1.148 ± 0.177 #	77.6%	1.45 ± 0.28 ***	30.6%
MA	6	29.4 ± 1.3	0.055 ± 0.010	6	32.4 ± 0.5	1.076 ± 0.388 #	69.2%	1.26 ± 0.25 #	39.6%
HA	6	27.5 ± 3.8 *	0.055 ± 0.008	6	32.2 ± 0.9	0.876 ± 0.262 #	57.0%	1.03 ± 0.13 #	50.6%

Note: Compared to the NS group: * *p* < 0.05, *** *p* < 0.001, # *p* < 0.0001.

**Figure A2:**
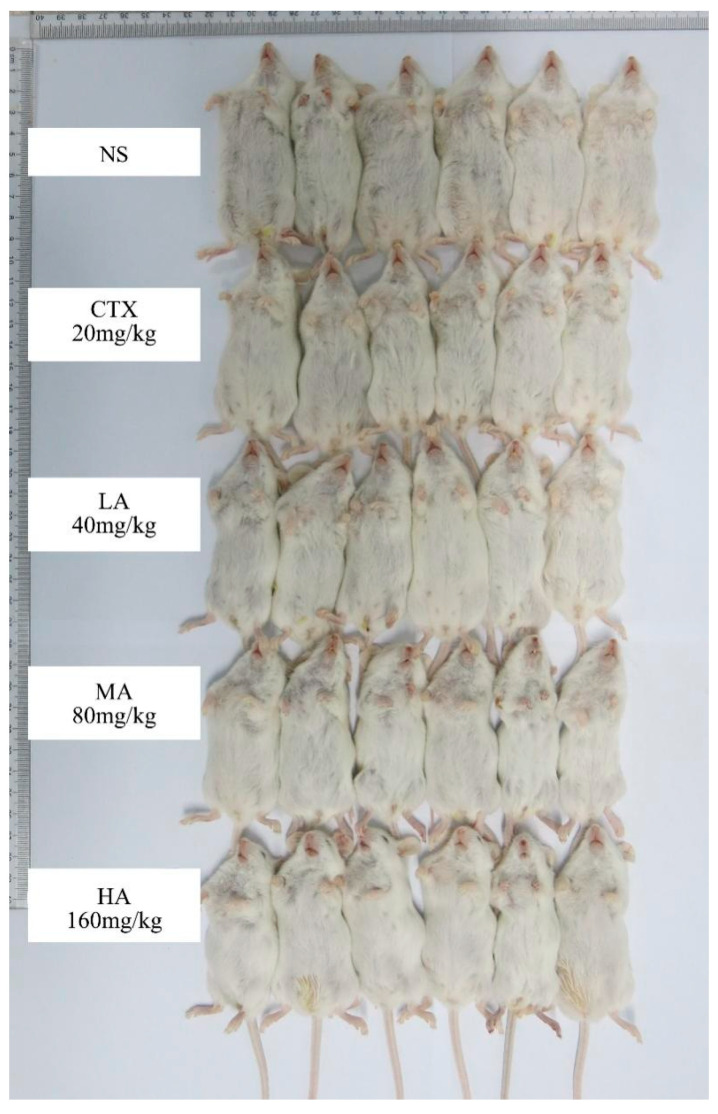
Experimental Grouping and Mouse Samples in the Study.

## Data Availability

The original contributions presented in this study are included in the article. Further inquiries can be directed to the corresponding author.

## Abbreviations

The following abbreviations are used in this manuscript:

AKT	Protein Kinase B
Annexin V-FITC/PI	Fluorescein Isothiocyanate Annexin V And Propidium Iodide
AP-1	Activator Protein 1
ARC	Arctigenin
AURKA	Aurora Kinase A
BCA	Bicinchoninic Acid Protein Assay
BP	Biological Process
BRAF	Serine/Threonine-Protein Kinase
CC	Cellular Component
CCNB1	G2/Mitotic-Specific Cyclin-B1
CCND1	G1/S-Specific Cyclin-D1
CCND2	G1/S-Specific Cyclin-D2
CDK1	Cyclin-Dependent Kinase 1
CDK2	Cyclin-Dependent Kinase 2
CDK4	Cyclin-Dependent Kinase 4
CMC-Na	Carboxymethylcellulose Sodium
Con	Blank Control Group (no treatment)
COU	Coulomb Force
CTX	Cyclophosphamide
EMT	Epithelial–Mesenchymal Transition
ErbB	Erythroblastic Leukemia Viral Oncogene Homolog
FoxO	Forkhead Box O
GAPDH	Glyceraldehyde-3-Phosphate Dehydrogenase
GM-CSF	Granulocyte-Macrophage Colony-Stimulating Factor
GO	Gene Ontology
GSK3B	Glycogen Synthase Kinase 3 Beta
HA	High-Dose Arctigenin Treated Group
HCC	Hepatocellular Carcinoma
HepG2	Human Hepatocellular Carcinoma Cell Line
HPLC	High Performance Liquid Chromatography
IARC	International Agency For Research On Cancer
IC50	Half Inhibitory Concentration
JAK-STAT	Janus Kinase-Signal Transducer And Activator Of Transcription
KEGG	Kyoto Encyclopedia Of Genes And Genomes
LA	Low-Dose Arctigenin Treated Group
MA	Medium-Dose Arctigenin Treated Group
MA + LY	Medium-Dose Arctigenin + Ly294002 Treated Group
MAP2K1	Mitogen-Activated Protein Kinase Kinase 1
MAPK	Mitogen-Activated Protein Kinases
MD	Molecular Dynamics
Met	Positive Control Group (Treated with Metformin)
MF	Molecular Function
MM	Molecular Interaction Force
mTOR	Serine/Threonine-Protein Kinase
NC	Network Centrality
NF-κB	Nuclear Factor Kappa-Light-Chain-Enhancer Of Activated B Cells
NS	Model Control
PB	Polar Solvation Energy
PI3K	Phosphoinositol-3 Kinase
PIK3CA	Phosphatidylinositol-4,5-Bisphosphate 3-Kinase Catalytic Subunit Alpha
PIK3R1	Phosphoinositol-3-Kinase Regulatory Subunit Alpha
PPI	Protein–Protein Interaction
RAF1	Rapidly Accelerated Fibrosarcoma Kinase
RMSD	Root Mean Square Deviation
ROS	Reactive Oxygen Species
RTV	Relative Tumor Volume
SA	Nonpolar Solvation Energy
Sim	Positive Control Group (Treated with Simvastatin)
SMILES	Simplified Molecular-Input Line-Entry System
SMM	Saussurea medusa Maxim.
STAT3	Signal Transducer And Activator Of Transcription 3
T/C	Relative Tumor Proliferation Rate
TACE	Transcatheter Arterial Chemoembolization
TGF-β	Transforming Growth Factor Beta
TSLP	Thymic Stromal Lymphopoietin
TV	Tumor Volume
-TΔS	Entropy Change
VDW	Van Der Waals Force
ΔG	Binding Free Energy
ΔH	Enthalpy Change

## Appendix A

**Table A1:** Effects of the Tested Samples on the Body Weight of Mice with H22 Cells.

Group	1st Measurement	2nd Measurement	3rd Measurement	4th Measurement	5th Measurement	6th Measurement	7th Measurement
NS	29.4 ± 1.0	30.1 ± 1.1	30.8 ± 1.1	31.9 ± 1.0	33.1 ± 1.2	33.4 ± 1.2	33.6 ± 1.2
CTX	29.5 ± 0.8	30.0 ± 0.7	30.2 ± 0.7	31.4 ± 1.4	32.9 ± 1.2	33.1 ± 1.2	33.3 ± 1.1
LA	28.7 ± 1.7	29.4 ± 1.4	29.8 ± 1.3	30.8 ± 1.3	31.2 ± 1.1 *	31.4 ± 1.0 *	31.6 ± 0.8 *
MA	29.4 ± 1.3	29.9 ± 1.2	30.3 ± 1.2	31.3 ± 0.7	31.9 ± 0.6	32.2 ± 0.6	32.4 ± 0.5
HA	27.5 ± 3.8 *	29.0 ± 2.1	29.9 ± 1.4	31.2 ± 1.2	32.1 ± 1.0	32.1 ± 1.0	32.2 ± 0.9

Note: *x* ± SD, *n* = 6, Unit: g. Compared with the NS group: * *p* < 0.05.

**Table A2:** Effects of the Tested Samples on Tumor Volume in Mice with H22 Cells.

Group	1st Measurement	2nd Measurement	3rd Measurement	4th Measurement	5th Measurement	6th Measurement	7th Measurement
NS	0.072 ± 0.021	0.132 ± 0.023	0.355 ± 0.068	0.689 ± 0.064	1.028 ± 0.060	1.516 ± 0.134	1.944 ± 0.210
CTX	0.052 ± 0.010	0.082 ± 0.020	0.137 ± 0.032	0.261 ± 0.083 #	0.357 ± 0.124 #	0.524 ± 0.273 #	0.626 ± 0.312 #
LA	0.053 ± 0.010	0.099 ± 0.034	0.270 ± 0.051	0.495 ± 0.115	0.695 ± 0.140 **	1.008 ± 0.172 #	1.148 ± 0.177 #
MA	0.055 ± 0.010	0.098 ± 0.028	0.264 ± 0.066	0.493 ± 0.164	0.679 ± 0.199 **	0.980 ± 0.372 #	1.076 ± 0.388 #
HA	0.055 ± 0.008	0.075 ± 0.019	0.212 ± 0.070	0.387 ± 0.150 **	0.554 ± 0.220 #	0.723 ± 0.248 #	0.876 ± 0.262 #

Note: *x* ± SD, *n* = 6, Unit: g. Compared with the NS group: ** *p* < 0.01, # *p* < 0.0001.

**Table A3:** Effects of the Tested Samples on Relative Tumor Volume in Mice with H22 Cells.

Group	2nd Measurement	3rd Measurement	4th Measurement	5th Measurement	6th Measurement	7th Measurement
NS	1.88 ± 0.23	5.31 ± 1.94	10.02 ± 2.02	15.08 ± 3.49	22.03 ± 4.28	28.20 ± 5.62
CTX	1.59 ± 0.33	2.70 ± 0.75	5.08 ± 1.48 *	6.94 ± 2.09 #	9.92 ± 3.69 #	11.89 ± 4.24 #
LA	1.81 ± 0.38	5.10 ± 0.80	9.36 ± 1.91	13.17 ± 2.46	19.15 ± 3.36	21.89 ± 4.02 **
MA	1.77 ± 0.27	4.81 ± 0.71	8.88 ± 1.90	12.36 ± 2.17	17.72 ± 4.51 *	19.50 ± 4.65 #
HA	1.36 ± 0.19	3.82 ± 1.01	7.02 ± 2.54	10.15 ± 4.12 *	13.26 ± 4.62 #	16.07 ± 4.90 #

Note: *x* ± SD, *n* = 6, Unit: g. Compared with the NS group: * *p*< 0.05, ** *p*< 0.01, # *p* < 0.0001.
